# ECOG and BMI as preoperative risk factors for severe postoperative complications in ovarian cancer patients: results of a prospective study (RISC-GYN—trial)

**DOI:** 10.1007/s00404-021-06116-5

**Published:** 2021-06-24

**Authors:** Melisa Guelhan Inci, Julia Rasch, Hannah Woopen, Kristina Mueller, Rolf Richter, Jalid Sehouli

**Affiliations:** Charité – Universitätsmedizin Berlin, corporate member of Freie Universität Berlin, Humboldt-Universität zu Berlin, and Berlin Institute of Health, Campus Virchow Klinikum, Department of Gynecology with Center for Oncological Surgery, European Competence Center for Ovarian Cancer, Berlin, Germany

**Keywords:** Ovarian cancer, Performance status, Body mass index, Comorbidity, Gynecology, Surgery, Postoperative complications

## Abstract

**Background:**

Accompanying co-morbidities in patients with ovarian cancer are of major relevance for scheduling debulking surgery, especially in the anesthesiological consultations. Aim of this study was to evaluate the impact of co-morbidities and patient characteristics on postoperative complications.

**Methods:**

Patients undergoing maximal cytoreductive surgery were prospectively enrolled from October 2015 to January 2017. Various variables were recorded, such as the Charlson comorbidity index, Eastern cooperative oncology group scale of performance status (ECOG PS) and the American society of anesthesiologists physical status classification system (ASA PS). Surgical complications were graded using the Clavien–Dindo criteria. Logistic regression models were used to analyze risk factors for severe postoperative complications.

**Results:**

Of 106 enrolled patients, 19 (17.9%) developed severe postoperative complications grade ≥ IIIb according to Clavien–Dindo criteria. In the multivariable regression analysis impaired (ECOG PS) > 1 (odds ratio OR) 13.34, 95% confidence interval (CI) 1.74–102.30, *p* = 0.01), body mass index (BMI) > 25 kg/m^2^ (OR 10.48, 95% CI 2.38–46.02, *p* = 0.002) along with the use of intraoperative norepinephrine > 0.11 µg/kg/min (OR 4.69, 95% CI 1.13–19.46, *p* = 0.03) and intraoperative fresh frozen plasma (FFP) > 17 units (OR 4.11, 95% CI 1.12–15.14, *p* = 0.03) appeared as significant predictors of severe postoperative complications.

**Conclusion:**

We demonstrated that neither the presence of a certain comorbidity nor the summation of the co-morbidities were associated with adverse outcome. Patient characteristics, such as ECOG PS > 1 and obesity (BMI > 25 kg/m^2^), are highly predictive factors for severe postoperative complications. The analysis of intraoperative data showed that the need for more than > 0.11 µg/kg/min of norepinephrine and transfusions of FFPs more than 17 units were strongly associated with severe postoperative complications.

**Supplementary Information:**

The online version contains supplementary material available at 10.1007/s00404-021-06116-5.

## Background

Complete cytoreduction is one of the main prognostic factors for patients with ovarian cancer [1–3]. To achieve complete macroscopic tumor resection, extensive multi-visceral procedures are performed [4]. Severe complications can be the result of complex procedures. These complications can result in prolonged recovery periods and delays in the initiation of adjuvant therapy with consequent worsened overall outcome [4, 5]. When alternate strategies such as neoadjuvant chemotherapy are discussed, the individual evaluation of patients is crucial [6–8]. Accompanying co-morbidities in patients with ovarian cancer are of great importance in the pre-surgical anesthesiological consultation for the debulking operation [9]. Also, elements, such as age, body mass index (BMI), American society of anesthesiologists physical status classification system (ASA PS), preoperative albumin, Fédération Internationale de Gynécologie et d’Obstétrique (FIGO) stage, and surgical complexity, were found to hold a significant level of predictive ability [10–14].

The aim of this study was to evaluate potential predictive markers for severe postoperative complications in ovarian cancer surgery, such as co-morbidities and further clinical characteristics.

## Methods

### Study design and patient population

**Fig. 1:**
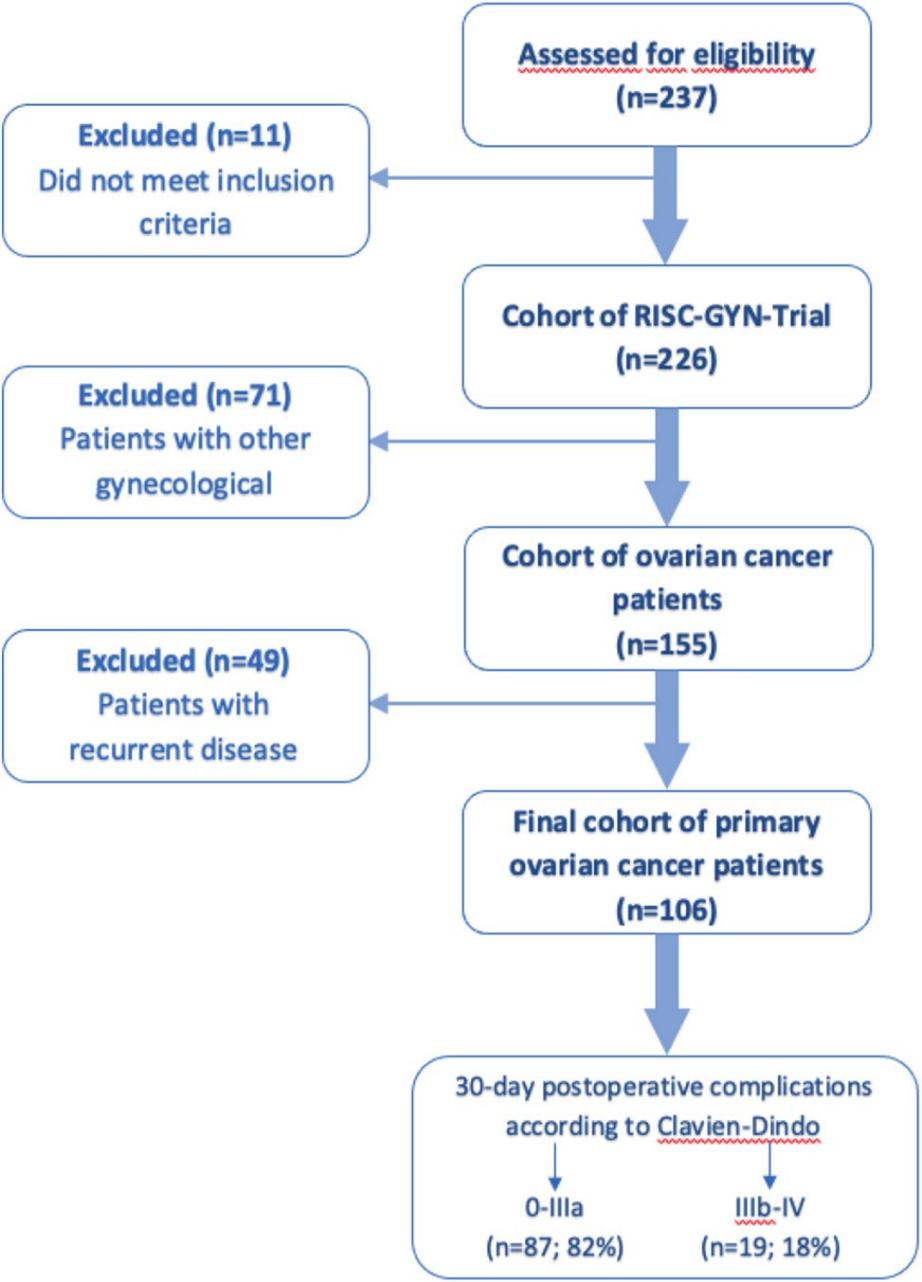
Consort diagram

This analysis is part of the prospective study named “RISC-GYN—trial”. The study was designed to explore predictive markers for severe postoperative complications in gynecologic cancer patients. Out of this cohort with gynecological cancer patients, we selected 155 ovarian cancer patients as the largest entity within the RISC-GYN—trial. We later excluded patients with recurrent disease. See Fig. 1 for consort diagram. Ethical approval was received from the local Ethics Committee (Charité–Universitätsmedizin Berlin) with the approval ID EA2/122/15. Data have been collected prospectively from October 2015 to January 2017. All surgeries were performed by specialized gynecologic oncology surgeons. Inclusion criteria were age above 18 years, histologically confirmed malignancy or a high suspicion of a gynecologic malignancy arising from imaging and laboratory results, and surgery with an expected minimal duration of 60 min. Written consent was required from all patients. Assessment consisted of a thorough medical history of all patients including co-morbidities and medications, as well as height and weight. Single-item questions were asked concerning sociodemographic, lifestyle and physical activity. Laboratory values were derived from preoperative routinely performed blood tests.

The Charlson comorbidity index was calculated for each patient as previously published by Charlson et al. [15]. By adding one further point per decade, starting at 50–59, the age-adjusted Charlson comorbidity index was calculated [16]. To define each patient’s performance status, ECOG PS and ASA PS were recorded by gynecologists and anesthesiologists prior to surgery [17–19].

Information on performed operative procedures, perioperative anesthesiologic parameters and tumor dissemination pattern was documented intraoperatively. The intraoperative mapping of ovarian cancer (IMO) was applied for exact tumor documentation [20].

Each patient was visited systematically in a daily schedule until the end of their hospital stay and was followed up via phone after 3 months post surgery. Our primary outcome of interest was 30-day postoperative complications Clavien–Dindo classification grade IIIb, IV and V. Clavien–Dindo classification grade IIIb complications require an intervention under general anesthesia [21]. See Supplementary 1 for detailed explanation. According to the severity of their postoperative complications, patients were divided into two cohorts: non- and mild postoperative complications (grade 0–IIIa) and severe postoperative complications (grade IIIb–V).

### Statistical analysis

Where appropriate the Fisher’s exact test for dichotomous variables, Kendall’s tau b for ordinal variables, the chi test for nominal variables and Kruskal–Wallis test or Mann–Whitney test for continuous variables were performed to evaluate comparisons between groups.

**Fig. 2:**
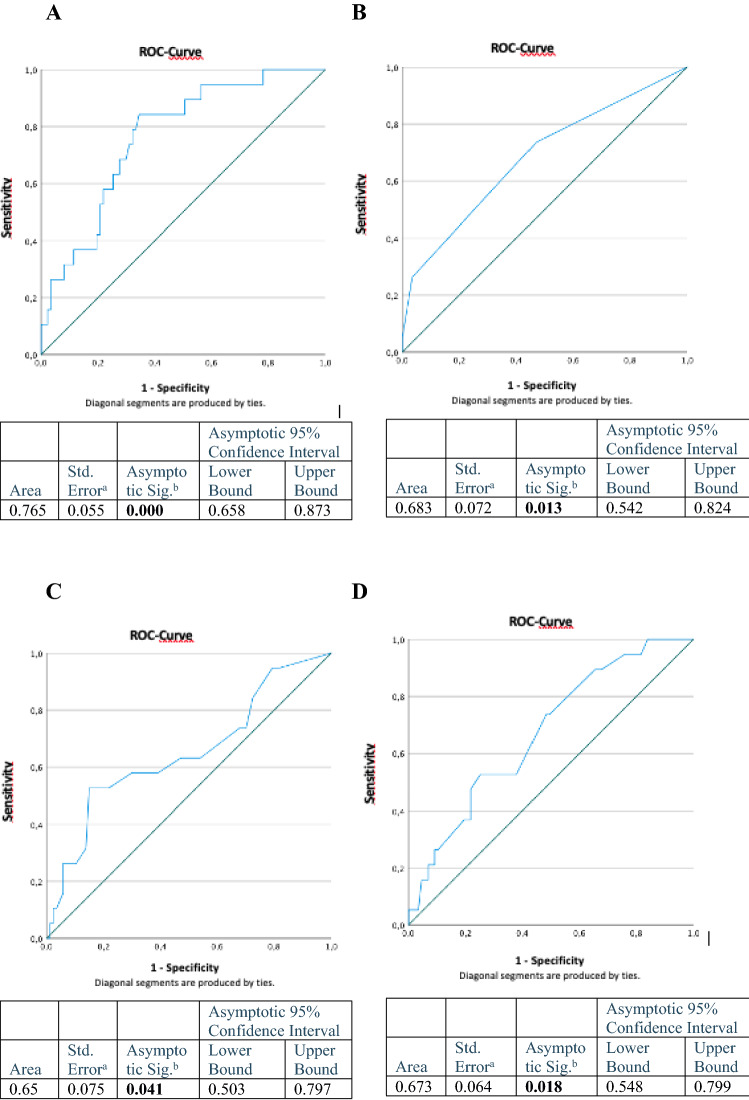
ROC analysis to define cut-offs for **A** BMI, **B** ECOG PS, **C** number of transfused FFP and, **D** highest intraoperative need for norepinephrine for predicting severe postoperative complications

To assess the predictive accuracy of continuous variables for distinguishing patients with severe postoperative complications from those without severe postoperative complications and to determine cut-offs, receiver-operator characteristics (ROC) curve analyses were used (see Fig. 2). Logistic regression analysis was performed to obtain crude and adjusted odds ratios (ORs) with corresponding 95% confidence interval (95% CI). Multivariable logistic regression analyses were executed stepwise with *p*_in_ = 0.05 and *p*_out_ = 0.10. Excluded from the multivariable analyses were cases with missing values. For statistical analysis, IBM^®^ SPSS^®^ Statistics 25 (SPSS Inc. an IBM Company, Chicago, IL, USA) was used and statistical significance was defined as *p* < 0.05 without alteration for multiple comparisons.

## Results

A total of 106 women undergoing surgery for ovarian cancer were enrolled in the study. Baseline characteristics of the study cohort are presented in Table 1.

**Table 1:** Baseline Characteristics

Characteristics	Total *n* = 106	Clavien–Dindo classification grade 0–IIIa *n* = 87	Clavien–Dindo classification grade ≥ IIIb *n* = 19	*p* value
	(range or %)	*n* (range or %)	*n* (range or %)	
Median age in years	57 (18–87)	55 (18–86)	63 (31–87)	0.2
Age ≥ 65 years	42 (39.6)	32 (36.8)	10 (52.6)	0.2
Age ≥ 70 years	28 (26.4)	21 (24.1)	7 (36.8)	0.3
Underweight (BMI < 20 kg/m^2^)	15 (14.2)	15 (17.2)	0	< 0.001
Normal (BMI 20–25 kg/m^2^)	43 (40.6)	40 (46.0)	3 (15.8)	
Overweight (BMI 25–30 kg/m^2^)	28 (26.4)	19 (21.8)	9 (47.4)	
Obese (BMI > 30 kg/m^2^)	20 (18.9)	13 (14.9)	7 (36.8)	
Preoperative albumin < 35.6 g/l	15 (15.6)	9 (11.5)	6 (33.3)	0.02
Preoperative potassium < 3.7 mmol/l	12 (11.4)	7 (8.1)	5 (26.3)	0.02
Charlson comorbidity index ≥ 1	32 (30.2)	20 (23.0)	12 (63.2)	0.001
Hypertension	34 (32.1)	27 (31.0)	7 (36.8)	0.6
Diabetes mellitus type I and type II	9 (8.5)	5 (5.7)	4 (21.1)	0.03
Polypharmacy (> 5 medications)	21 (19.8)	13 (14.9)	8 (42.1)	0.007
Smoking daily	24 (22.5)	21 (24.1)	3 (15.8)	0.4
Alcohol consumption daily	13 (12.3)	11 (12.6)	2 (10.5)	0.8
Performance status				
ASA PS > 2	29 (27.6)	21 (24.1)	8 (44.4)	0.08
ECOG PS > 1	8 (7.5)	3 (3.4)	5 (26.3)	0.001
FIGO stage				0.6
I–II	17 (16.2)	15 (17.4)	2 (10.6)	0.4
III–IV	88 (83.8)	71 (82.5)	17 (89.6)	
High-grade	91 (92.9)	75 (92.6)	16 (94.1)	0.2
Low-grade	7 (7.1)	6 (7.4)	1 (5.9)	
Neoadjuvant chemotherapy	11	9	2	1.0
Presence of ascites	59 (59.0)	44 (54.3)	15 (78.9)	0.4

*BMI* body mass index, *ASA PS* American society of anesthesiologists physical status classification system, *ECOG PS* eastern cooperative oncology group scale of performance status, *FIGO* fédération internationale de gynécologie et d’obstétrique, *CA* cancer antigen

Overall, 19 patients (17.9%) experienced complications Clavien–Dindo Classification grade ≥ IIIb and one of them (0, 9%) died within 30 days of surgery. The most common complications were urinary tract infections (*n* = 14, 13%), postoperative pleural effusions (*n* = 31, 29%), anastomotic insufficiency (*n* = 9, 8%), wound dehiscence (*n* = 5, 4%), peritonitis (*n* = 5, 4%), and thromboembolic events (*n* = 5, 4%). Table 2 shows the complications that occurred. The most common co-morbidities were arterial hypertension (*n* = 34, 32%), polyneuropathy (*n* = 23, 22%), chronic pulmonary disease (*n* = 10, 9%) and diabetes mellitus type I and type II (*n* = 9, 8%). Cardiovascular disease (*n* = 35, 37%) was defined as current or condition after myocardial infarction, heart failure, peripheral vascular disease, cerebrovascular disease, cardiac arrhythmias and atrial fibrillation. See Table 3 for further details of the distribution of co-morbidities.

**Table 2:** Types and frequency of postoperative complications

Type	*n* (%)
Wound dehiscence	5 (4.7)
Anastomotic insufficiency	9 (8.5)
Thromboembolic events	5 (4.7)
Ileus	3 (2.8)
Organ failure	1 (0.9)
Sepsis	4 (3.8)
Peritonitis	5 (4.7)
Fistula	1 (0.9)
Intestinal perforation	2 (1.9)
Delir	4 (3.8)
Neurological disorder	5 (4.7)
Postoperative pleural effusions	31 (29.3)
Pneumonia	5 (4.7)
Pneumothorax	3 (2.8)
Urinary tract infections	14 (13.2)

**Table 3:** Distribution of co-morbidities according to the Charlson comorbidity index and co-morbidities not included in the Charlson comorbidity index

Charlson comorbidity index	*n* (%)
Diabetes mellitus without end-organ damage	9 (8.5)
Cerebrovascular disease	2 (1.9)
Myocardial infarction	4 (3.8)
Congestive heart failure	5 (4.7)
Peripheral vascular disease	2 (1.9)
Dementia	0
Chronic pulmonary disease	10 (9.4)
Connective tissue disease	4 (3.8)
Peptic ulcer disease	3 (2.8)
Mild liver disease	1 (0.9)
Diabetes mellitus with end-organ damage	0
Moderate/severe renal disease	2 (1.9)
Hemiplegia	0
Solid tumor without metastasis	0
Leukemia	0
Lymphoma	0
Moderate/severe liver disease	1 (0.9)
Metastatic solid tumor	0
Acquired immune deficiency syndrome	0

Comorbidities not included in Charlson comorbidity index	*n* (%)
Hypertension	34 (32.1)
Polyneuropathy	23 (14.8)
Lymphedema	3 (2.8)
Cardiac arrhythmias	14 (13.2)
Atrial fibrillation	8 (7.5)

The patients with serious postoperative complications had significantly more co-morbidities (Charlson comorbidity index ≥ 1; 63 vs. 23%; *p* = 0.001), a higher rate of polypharmacy (> 5 medications; 42 vs. 15%; *p* = 0.007), diabetes (21 vs. 6%; *p* = 0.03), compared to patients without or with less severe complications. They showed significantly more often hypokalemia (26 vs. 8%; *p* = 0.02), hypoalbuminemia (33 vs. 12%; *p* = 0.02) and a high level of CA 125 (> 500 U/ml) (28 vs. 7%; *p* = 0.005). Their ECOG PS was more often impaired (26 vs. 3%; *p* = 0.001) age as a continuous variable (OR 1.91, 95% CI 0.70–5.19, *p* = 0.2) and age ≥ 70 years (OR 1.83, 95% CI 0.64–5.26, *p* = 0.3) showed no association with severe postoperative complications.

### Analysis of association of preoperative patient-related characteristics and severe postoperative complications (≥ grade IIIb) by Clavien–Dindo classification

An ASA PS > 2 and ECOG PS > 1 were associated with severe complications (OR 2.51; 95% CI 0.88–7.20; *p* < 0.09) and (OR 10.0; 95% CI 2.15–46.61; *p* = 0.003), respectively.

Univariate logistic regression showed further that an increased number of co-morbidities (Charlson comorbidity index ≥ 1) was significantly associated with severe postoperative complications (OR 5.74, 95% CI 2.00–16.54, *p* = 0.001). The age-adjusted Charlson comorbidity index showed significance at a cut-off point of > 2 (OR 5.08, 95% CI 1.74–14.83, *p* = 0.003).

Cardiovascular disease (OR 3.79, 95% CI 1.29–11.12, *p* = 0.02) and diabetes (OR 4.37, 95% CI 1.05–18.19, *p* = 0.04), bowel obstruction symptoms (OR 8.00, 95% CI 1.23–51.56, *p* = 0.03) and the simultaneous intake of more than five medications (OR 4.14, 95% CI 1.40–12.25, *p* = 0.01) were significantly related in univariate logistic regression, whereas hypertension was not. Liver disease, chronic pulmonary disease and renal disease did not show correlation to severe postoperative complications. Hypokalemia (< 3.7 mmol/l) (OR 4.03, 95% CI 1.12–14.50, *p* = 0.03), a decreased INR (≤ 0.9) (OR 9.84, 95% CI 1.71–56.68, *p* = 0.01) and hyperbilirubinemia (> 0.46 mg/dl) (OR 3.62, 95% CI 0.98–13.42, *p* = 0.05) were associated with severe postoperative complications.

Overweight patients with a BMI > 25–30 kg/m^2^ were 8 times more likely to develop complications (OR 8.68, 95% CI 2.13–35.46, *p* = 0.003) and obese patients with a BMI above 30 kg/m^2^ were up to 9 times more likely to develop severe postoperative complications (OR 9.87, 95% CI 2.24–43.43, *p* = 0.002).

All the preoperative variables were included in the stepwise logistic regression. ECOG PS > 1 (OR 8.87, 95% CI 1.34–58.75, *p* = 0.02) showed the greatest correlation with severe postoperative complications. Overweight and obesity (BMI > 25 kg/m^2^, OR 8.54, 95% CI 1.82–31.37, *p* < 0.005) were highly predictive as well. Furthermore, the Charlson comorbidity index (OR 3.33, 95% CI 1.03–10.81, *p* = 0.05) was an independent predictive parameter. The age-adjusted Charlson comorbidity index and all the assessed co-morbidities lost significance in multivariate analysis. See Table 4 for detailed information of multivariate analysis for preoperative variables.

**Table 4:** Univariate analysis and multivariate analysis of preoperative factors related to severe postoperative complications

Patient demographics	Univariate analysis unadjusted OR (95% CI)	*p* value	Multivariate analysis adjusted OR (95% CI)	*p* value
Age (continuous variable)	1.91 (0.70–5.19)	0.2		
Age ≥ 70 years	1.83 (0.64–5.26)	0.3		
Alcohol consumption daily	0.81 (0.17–4.01)	0.8		
Smoking daily	0.59 (0.16–2.22)	0.4		
Performance status				
ASA PS > 2	2.51 (0.8–7.20)	0.09		
ECOG PS > 1	10.0 (2.15–46.61)	0.003	8.87 (1.34–58.75)	0.02
Comorbidities				
Charlson comorbidity index ≥ 1	5.74 (2.00–16.54)	0.001	3.33 (1.03–10.81)	0.05
Age-adjusted Charlson comorbidity index > 2	5.08 (1.74–14.83)	0.003		
Polypharmacy (> 5 medications)	4.14 (1.40–12.25)	0.01		
Bowel obstruction symptoms	8.00 (1.23–51.56)	0.03		
Diabetes mellitus type I and II	4.37 (1.05–18.19)	0.04		
Cardiovascular disease	3.79 (1.29–11.12)	0.02		
Renal disease	1.09 (0.12–10.13)	1.0		
Chronic pulmonary disease	2.14 (0.50–9.18)	0.3		
Liver disease	4.78 (0.29–80.0)	0.3		
Hypertension	1.30 (0.46–3.66)	0.6		
Thromboembolic event	0.86 (0.18–4.15)	1.0		
Polyneuropathy	0.75 (0.90–6.62)	0.8		
BMI (kg/m^2^) categorical				
< 20	0.00 (0.00–0.00)	1.00		
26–30 overweight	8.68 (2.13–35.46)	0.003		
> 30 obese	9.87 (2.24–43.43)	0.002	8.54 (1.82–31.37)	0.005
Laboratory variables				
Potassium < 3.7 mmol/l	4.03 (1.12–14.50)	0.03		
INR ≤ 0.9	9.84 (1.71–56.68)	0.01		
Creatinine > 0.75 mg/dl	2.01 (0.74–5.47)	0.17		
Albumin < 35.6 g/l	3.83 (1.15–12.74)	0.03		
GFR < 81.5 ml/min	0.43 (0.16–1.17)	0.1		
Hemoglobin < 12.25 g/dl	0.37 (0.99–1.37)	0.1		
Sodium < 137 mmol/l	1.43 (0.35–5.77)	0.6		
ALT > 35 U/l	1.65 (0.39–6.97)	0.5		
AST > 35 U/l	1.34 (0.38–4.74)	0.7		
Bilirubin > 0.46 mg/dl	3.62 (0.98–13.42)	0.05		

*ASA PS* American society of anesthesiologists physical status classification system, *ECOG PS* eastern cooperative oncology group scale of performance status, *BMI* body mass index, *INR* international normalized ratio, *GFR* glomerular filtration rate, *ALT* alanine transaminase, *AST* aspartate transaminase

### Association of intraoperative data, tumor-related data and severe postoperative complications (≥ grade IIIb) by Clavien–Dindo classification

The surgical procedures of special interest included bowel resection with resection of the large intestine (*n* = 56, 53%), the small intestine (*n* = 17, 16%), upper abdominal surgery (*n* = 36, 34%) and pelvic and/or paraaortical lymphonodectomy (*n* = 77, 72%). In univariate analysis, most of the surgical procedures, prolonged duration of surgery, intraoperative hypothermia (< 36 °C) and most of the tumor-related characteristics did not show significant correlation with complications.

Only the use of more than 0.11 µg/kg/min norepinephrine (OR 3.00, 95% CI 0.99–9.05, *p* = 0.05), the use of fresh frozen plasma (OR 4.26, 95% CI 1.51–12.04, *p* = 0.006) and a level of CA 125 > 500 U/ml (OR 2.98, 95% CI 1.04–8.51, *p* = 0.04) showed significant association.

The intraoperative and tumor-related parameters were also analyzed in a stepwise logistic regression. Only the use of more than 17 intraoperative fresh frozen plasma units (OR 5.93, 95% CI 1.70–20.61, *p* = 0.005) and the pelvic and/or para-aortic lymphadenectomy (OR 3.43, 95% CI 1.04–11.30, *p* = 0.04) remained significantly associated with severe postoperative complications.

See Table 5 for further details. See Fig. 2 for the ROC-analysis that was used to define cut-offs for prolonged surgery duration and highest intraoperative need for norepinephrine for predicting severe postoperative complications.

**Table 5:** Univariate analysis and multivariate analysis of intraoperative data and tumor-related characteristics

30 day-severe complications (Clavien–Dindo IIIb–V)						*n* = 29 *p* value
Characteristics	*n*	%	Univariate analysis	*p* value	Multivariate analysis	
			Unadjusted OR (95% CI)		Adjusted OR (95% CI)	
Anaestesiological data						
Surgery duration > 255 min	76	71.7	1.60 (0.48–5.28)	0.4		
Norepinephrine > 0.11 µg/kg/min	56	52.8	3.00 (0.99–9.05)	0.05		
Intraoperative FFP > 17	23	21.7	4.26 (1.51–12.04)	0.006	5.93 (1.70–20.61)	0.005
Blood transfusion > 1	48	45.3	2.43 (0.87–6.77)	0.09		
Hypothermia (intraoperative temperature < 36 °C)	32	30.2	0.31 (0.06–1.73)	0.2		
Operative data						
Pelvic and/or para-aortic lymphadenectomy	77	72.6	2.29 (0.81–6.43)	0.1	3.43 (1.04–11.30)	0.04
Upper abdominal surgery	36	34.0	1.53 (0.56–4.32)	0.4		
Large bowel resection	56	52.8	1.83 (0.60–5.55)	0.3		
Small bowel resection	17	16.0	0.98 (0.25–3.81)	1.0		
Peritoneal carcinomatosis	83	78.3	2.71 (0.58–12.68)	0.2		
Neoadjuvant chemotherapy	11	10.4	1.02 (0.20–5.15)	1.0		
Tumor-related characteristics						
No residual disease	67	63.2				
Residual disease	37	34.9	1.40 (0.51–3.88)	0.5		
FIGO stage						
I	11	10.4				
II	6	5.7	2.00 (1.00–39.08)	0.7		
III	65	61.3	2.26 (0.26–19.42)	0.5		
IV	23	21.7	2.78 (0.28–27.21)	0.4		
Low-grade	7	6.6				
High-grade	91	85.8	1.35 (0.28–6.47)	0.7		
Presence of ascites	70	66.0	3.15 (0.96–10.33)	0.06		

*FFP* fresh frozen plasma, *FIGO* Fédération Internationale de Gynécologie et d’Obstétrique, *CA* cancer antigen

### Multivariate analysis of preoperative, intraoperative and tumor-related parameters and severe postoperative complications (≥ grade IIIb) by Clavien–Dindo classification

Ultimately, to all significant factors from the preoperative analysis, the intraoperative and tumor-related parameters were added into stepwise logistic regression. Table 6 shows BMI > 25 kg/m^2^ (OR 10.48, 95% CI 2.38–46.02, *p* = 0.002), ECOG PS > 1 (OR 13.34, 95% CI 1.74–102.30, *p* = 0.01), the use of more than > 0.11 µg/kg/min norepinephrine (OR 4.69, 95% CI 1.13–19.46, *p* = 0.03) and the use of more than 17 intraoperative FFP units (OR 4.11, 95% CI 1.12–15.14, *p* = 0.03) predictive for severe postoperative complications.

**Table 6:** Multivariable analysis of patient-related, tumor-related and intraoperative factors for severe postoperative complications

	Adjusted OR (95% CI)	*p* value
Overweight and obesity (BMI > 25 kg/m^2^)	10.48 (2.38–46.02)	0.002
ECOG performance status > 1	13.34 (1.74–102.30)	0.01
Intraoperative fresh frozen plasma > 17	4.11 (1.12–15.14)	0.03
Norepinephrine > 0.11 µg/kg/min	4.69 (1.13–19.46)	0.03

*ECOG PS* Eastern cooperative oncology group scale of performance status

## Discussion

Several studies report an association between co-morbidities and clinical outcome but most of them are performed retrospectively and heterogeneous patient populations limit their interpretation [14, 22]. Therefore, we conducted this prospective study in patients with ovarian cancer.

We screened systematically all co-morbidities and several preoperatively assessed patient-related characteristics, as well as intraoperative anaesthesiological, surgical and tumor-related parameters in this prospective study to assess their impact on 30 days’ postoperative complications in ovarian cancer patients. In the first part of the data analysis, we demonstrated that preoperative patient-related characteristics, such as ECOG PS > 1, obesity and the Charlson comorbidity index, are predictive factors for postoperative complications. In the second part, the analysis of intraoperative data showed the performance of lymphadenectomy and the high need for fresh frozen plasma transfusions to be strongly associated with severe postoperative complications.

The combined evaluation of preoperative and intraoperative factors showed ECOG PS > 1, overweight and obesity/a BMI > 25 kg/m^2^, high need of intraoperative FFP and norepinephrine are associated with severe postoperative complications.

The age of the patients showed no association with complications, not even for patients above the age of 70 year. Other studies showed that older patients are at increased risk of surgical morbidity, but if they are able to tolerate aggressive surgical management, the benefit was equivalent to that of younger women [23]. In contrast to our results, age and stage were associated with increased risk for 30-day morbidity and mortality in other studies. However, these studies included only advanced-stage ovarian cancer patients and focused on the elderly [24, 25].

Previous studies have identified the ASA PS and ECOG PS as good tools for outcome prediction in cancer patients [3, 10, 23, 26]. Our results suggest that the ECOG PS is more relevant in oncologic surgery than the ASA PS, evaluating how independently a patient can manage everyday life rather than focusing on systemic diseases. Looking further to evaluate co-morbidities and organ dysfunction affecting the surgical outcome, none of the co-morbidities remained an independent predictor for postoperative complications in multivariate analysis. Our findings suggest that even multi-morbid patients do not require less aggressive surgical treatment plans.

Several publications focusing on ovarian cancer showed obesity as a highly predictive factor for postoperative complications [10, 26–32]. Extreme BMIs—both high and low, have been found to be associated with severe postoperative complications [10]. In our study, underweight was not predictive for postoperative complications, whereas obesity remained an independent predictor. This adds to an earlier prospective publication, which found frail patients to be obese rather than underweight and which stated that weight itself might not be a marker for physiologic reserve [13]. These patients often suffer from impaired wound healing and surgeons face more complex conditions in obese patients, as longer surgery duration is required to obtain equivalent surgical results [29, 31]. These findings are particularly important as obesity is becoming increasingly prevalent in our society.

In our analysis of intraoperative data, we intentionally did not group the surgical interventions as other studies did [12]. To examine the effect of each intervention on postoperative complications, we performed the stepwise logistic regression analysis with all surgical interventions of interest and only the lymphadenectomy showed significant correlation. This is in line with prior studies which discussed that patients with macroscopically complete resection did not benefit from systemic lymphadenectomy [33].

In our study, the use of high levels of norepinephrine and FFP correlated significantly with postoperative complications. The effect of vasoactive medication on gastrointestinal oxygen supply and microcirculatory blood flow is controversially discussed in several studies [34–37]. The exact interaction between catecholamines and simultaneously given anesthetics remains uncertain, as there are disparities of drug effect in different species and different gastrointestinal segments and experimental results cannot be extended into the clinical setting yet. However, if the microcirculation in the gastrointestinal in the gastrointestinal tract is reduced, this may cause impaired wound healing, especially in the anastomosis region. Similar to the report of other studies, the rate of anastomotic insufficiency in our cohort was 8.5% [38, 39]. Currently, the debate about the efficacy of FFP and its possible impact in cancer surgery is ongoing [40–43]. We assume that both, the need for norepinephrine and for FFP to stabilize hemodynamics during surgery are multi-factorially related and are indicators for poor general condition of the patient.

In this study, we screened all accompanying co-morbidities in patients with ovarian cancer systematically. Neither the summation of the co-morbidities assessed by the Charlson comorbidity index nor the presence of a certain comorbidity was associated with postoperative complications in the multivariate analysis of pre- and intraoperative parameters.

Our findings imply that the patient characteristics, such as ECOG performance status and BMI, could have greater effect on complications than the co-morbidities and the complex surgical interventions themselves.

There is a limitation noted for this study, as we included patients with different tumor stages and analyzed a heterogenous set of data. As our results are based on regression analysis, we cannot simply state causation, especially as the individual variables are influenced by each other. However, our findings contribute to a clearer picture of patients who will benefit from surgery.

The main strength of our study is, that it is a prospective study which examined predictive factors for postoperative complications as the primary endpoint. Using the Clavien–Dindo classification to grade postoperative complications made our results more objective as well as comparable and reproducible for future research. The homogeneous and very high quality of surgical treatment as well as daily visits of each patient lead to further improvement of reliability and validity of the acquired data.

## Conclusion

Surgeons should consider performance status rather than the sum of co-morbidities in future risk prediction. In this study, we identified potential parameters that can be preoperatively easily assessed and are feasible to collect in clinical routine. ECOG PS and BMI should be given more attention in clinical decision-making, treatment planning and patient counseling. A high intraoperative use of norepinephrine and FFP during cytoreductive surgery should lead to interdisciplinary communication between surgeons and anesthesiologists to give more awareness of the vulnerability of patients for complications.

## Supplementary Information

Below is the link to the electronic supplementary material.

Supplementary file1 (DOCX 16 kb)

## Data Availability

Raw data were generated at Charité Universitätsmedizin Berlin. Data are available upon reasonable request. All data relevant to the study are included in the article.
