# Supplementary material for: ECOG and BMI as preoperative risk factors for severe postoperative complications in ovarian cancer patients: results of a prospective study (RISC-GYN—trial)
Source: Arch Gynecol Obstet. 2021 Jun 24;304(5):1323–33. doi: 10.1007/s00404-021-06116-5 (PMC8490255; doi:10.1007/s00404-021-06116-5)
Supplement: Supplementary file 1 — Supplementary file1 (DOCX 16 kb) [file 404_2021_6116_MOESM1_ESM.docx]

**Supplementary 1: Surgical complications classified according to Clavien-Dindo[1]**

| **Grade Classification of surgical complications** | **Grade Classification of surgical complications** |
| --- | --- |
| I | Any divergence from the usual postoperative treatment path without the need for drug treatment or interventions. Drugs allowed: analgesics, antiemetics, electrolytes and diuretics. |
| II | Requiring further drug treatment, including parenteral nutrition and blood transfusions. |
| III | Surgical, radiological or endoscopic intervention |
| III a | Intervention without general anesthesia |
| III b | Intervention under general anesthesia. |
| IV | Life-threatening complications needing intensive care management (including central nervous system complications) |
| IV a | Dysfunction of a single organ |
| IV b | Dysfunction of multiple organs |
| V | Death |

1. Dindo D, Demartines N, Clavien P-A. Classification of Surgical Complications. Ann Surg. 2004 Aug;240(2):205–13.
